# Cloning, Prokaryotic Soluble Expression, and Analysis of Antiviral Activity of Two Novel Feline IFN-ω Proteins

**DOI:** 10.3390/v12030335

**Published:** 2020-03-19

**Authors:** Xiaona Wang, Fengsai Li, Meijing Han, Shuo Jia, Li Wang, Xinyuan Qiao, Yanping Jiang, Wen Cui, Lijie Tang, Yijing Li, Yi-Gang Xu

**Affiliations:** 1 Heilongjiang Key Laboratory for Animal Disease Control and Pharmaceutical Development, College of Veterinary Medicine, Northeast Agricultural University, Harbin 150030, China; xiaonawang0319@163.com (X.W.); Yilvwenrou@126.com (F.L.); h18246186256@163.com (M.H.); jiashuo0508@163.com (S.J.); wanglicau@neau.edu.cn (L.W.); qiaoxinyuan@neau.edu.cn (X.Q.); jiangyanping@neau.edu.cn (Y.J.); cuiwen@neau.edu.cn (W.C.); tanglijie@neau.edu.cn (L.T.); liyijing@neau.edu.cn (Y.L.); 2 Northeastern Science Inspection Station, China Ministry of Agriculture Key Laboratory of Animal Pathogen Biology, Harbin 150030, China

**Keywords:** novel feline interferon omega, gene cloning, molecular characteristics, soluble expression, antiviral activity

## Abstract

Cats are becoming more popular as household companions and pets, forming close relationships with humans. Although feline viral diseases can pose serious health hazards to pet cats, commercialized preventative vaccines are lacking. Interferons (IFNs), especially type I IFNs (IFN-α, IFN-β, and interferon omega (IFN-ω)), have been explored as effective therapeutic drugs against viral diseases in cats. Nevertheless, there is limited knowledge regarding feline IFN-ω (feIFN-ω), compared to IFN-α and IFN-β. In this study, we cloned the genes encoding feIFN-ωa and feIFN-ωb from cat spleen lymphocytes. Homology and phylogenetic tree analysis revealed that these two genes belonged to new subtypes of feIFN-ω. The recombinant feIFN-ωa and feIFN-ωb proteins were expressed in their soluble forms in *Escherichia coli,* followed by purification. Both proteins exhibited effective anti-vesicular stomatitis virus (VSV) activity in Vero, F81 (feline kidney cell), Madin–Darby bovine kidney (MDBK), Madin–Darby canine kidney (MDCK), and porcine kidney (PK-15) cells, showing broader cross-species antiviral activity than the INTERCAT IFN antiviral drug. Furthermore, the recombinant feIFN-ωa and feIFN-ωb proteins demonstrated antiviral activity against VSV, feline coronavirus (FCoV), canine parvovirus (CPV), bovine viral diarrhea virus (BVDV), and porcine epidemic diarrhea virus (PEDV), indicating better broad-spectrum antiviral activity than the INTERCAT IFN. The two novel feIFN-ω proteins (feIFN-ωa and feIFN-ωb) described in this study show promising potential to serve as effective therapeutic agents for treating viral infections in pet cats.

## 1. Introduction

As cats continue to become more popular as household companions and pets [1], a variety of viral infections pose a serious threat to felines, including a high incidence of feline leukemia virus (FeLV) [2], feline coronavirus (FCoV) [3], feline immunodeficiency virus (FIV) [4], and feline panleukopenia virus (FPV) [5]. Currently, the only preventative vaccines and therapeutic drugs available for use in pet cats have limited effectiveness. Nevertheless, interferons (IFNs) play an increasingly complementary role in antiviral therapy for virus-infected cats [6]. The main function of IFNs in the antiviral immune response is to induce the expression of antiviral proteins that then further activate antiviral, antiproliferative, and immunomodulatory cellular responses [7]. Generally, IFNs are classified into three subgroups: type I IFNs, type II IFNs, and type III IFNs. Type I IFNs, including IFN-α, IFN-β, IFN-ε, IFN-ω, IFN-κ, IFN-δ, IFN-τ, and IFN-ζ, play a direct role in antiviral immune responses [7,8]. Type II IFN, namely IFN-γ, is produced by T lymphocytes and natural killer cells in response to the recognition of infected cells [9]. Type III IFNs, including IFN-λ1, IFN-λ2, and IFN-λ3, regulate the antiviral immune response via a distinct receptor complex similar to type I IFNs [10,11].

Interferon omega (IFN-ω), which is produced primarily by leukocytes, displays similar structure and physicochemical characteristics to type I IFNs, and researchers have focused on developing therapeutic drugs based on feline IFN-ω (feIFN-ω) to treat viral infections in cats [6,12]. FeLV is an important pathogen in both domestic and wild cats that causes degenerative and immunosuppressive disorders, such as anorexia, apathy, cachexia, and progressive weakness [13,14]. Published research has demonstrated that feIFN-ω has the therapeutic potential to treat FeLV-infected cats [13,15], presumably by inhibiting the replication cycle of FeLV [16]. Several studies have revealed that recombinant feline IFN-ω (rfeIFN-ω) had statistically significant therapeutic effects on both FeLV infection and FeLV/FIV coinfection [13,17]. Currently, rfeIFN-ω, serving as an immunomodulator, is the first interferon licensed for use in cats to treat FeLV, FIV, and canine parvovirus infections [6,8,18]. Feline infectious peritonitis, which is triggered by feline infectious peritonitis virus (FIPV) (a virulent mutant of feline enteric coronavirus), is a lethal infectious disease in cats with still no effective drugs available for treatment [19,20,21]. Previous studies have demonstrated that rfeIFN-ω represents a therapeutic candidate for treating FIPV-infected cats [6]. In addition, data from several studies suggest that rfeIFN-ω can also exert protective effects on other viral infections, including bovine enterovirus (BEV), infectious bovine rhinotracheitis virus, bovine viral diarrhea virus (BVDV), vesicular stomatitis virus (VSV), pseudorabies virus (PRV), feline parvovirus, feline calicivirus, and feline coronavirus [6,18,22,23,24,25].

In this study, two novel genes encoding feIFN-ω, called feIFN-ωa and feIFN-ωb, were cloned from spleen lymphocytes of cats stimulated by VSV infection combined with poly(I:C) treatment. These genes were then expressed in their soluble forms in *Escherichia coli*. The molecular characteristics of feIFN-ωa and feIFN-ωb were analyzed, and the species-specific and broad-spectrum antiviral activities of both were evaluated in vitro. Our results suggest that the feIFN-ωa and feIFN-ωb proteins described in this study show promising potential to serve as effective therapeutic agents against feline viral infections.

## 2. Materials and Methods

### 2.1. Plasmids, Cells, and Viruses

The plasmid pMD19-T (simple) and the prokaryotic soluble expression plasmid pCold-TF were used in this study. African green monkey kidney (Vero) cells (ATCC CCL-81), Madin–Darby bovine kidney (MDBK) cells, porcine kidney (PK-15) cells, Madin–Darby canine kidney (MDCK) cells, and feline kidney (F81) cells were kept in our laboratory and maintained in Dulbecco’s Modified Eagle Medium (DMEM, Gibco, NY, USA) supplemented with 10% fetal bovine serum (FBS, Gibco, NY, USA) at 37 °C with 5% CO_2_. Feline coronavirus (FCoV) was propagated in F81 cells, porcine epidemic diarrhea virus (PEDV) was propagated in Vero cells, VSV was propagated in Vero cells, bovine viral diarrhea virus (BVDV) was propagated in MDBK cells, and canine parvovirus (CPV) was propagated in MDCK cells.

### 2.2. Animal and Ethics Statement

Healthy Dragon Li cats (*n* = 5) were purchased from a pet market in Harbin, China. Animal experiments were carried out in accordance with the recommendations in the Guide for the Care and Use of Laboratory Animals of the National Institutes of Health. The protocol (2018NEAU052x) was approved by the Ethical Committee for Animal Experiments at Northeast Agricultural University, Harbin, China (20 July 2018).

### 2.3. Cloning of feIFN-ω Genes

Confluent monolayers of F81 cells were infected with VSV at an MOI of 0.1 for 1 h at 37 °C in 5% CO_2_. After removal of the inoculum, the cells were washed and maintained in DMEM supplemented with 10% FBS (Gibco, Grand Island, NY, USA) until the cytopathic effect (CPE) was observed to exceed 90%, and then a freeze–thaw was performed. The supernatants were harvested and stored at −80 °C until required. The cats were intramuscularly injected daily with 2 mL of 100 TCID_50_ VSV combined with 500 μL of 1.0 mg/mL poly(I:C) for three consecutive days. On day 15 post-infection, the cats were euthanized, and the splenic lymphocytes were isolated aseptically and used for total RNA extraction with the TRIzol Total RNA Isolation kit (Invitrogen, Carlsbad, CA, USA) according to the manufacturer’s instructions. Subsequently, first-strand cDNA was reverse transcribed using a QuantiTect Reverse Transcription kit (QIAGEN, Hilden, Germany) according to the manufacturer’s instructions. The genes encoding feline IFN-ω were obtained by PCR amplification with the degenerate primer pair of F: 5′-GGTACCATGGCCCTCCTGCTCCCTCTRCT-3′ (containing a Kpn I restriction site) and R: 5′-GGATCCTCAYGATKACGCSSGRTCTCCAT-3′ (containing a BamH I restriction site), which were designed according to the gene sequences of known feIFN-ω published in the GenBank (accession number: DQ420220, DQ420221, DQ420222, DQ420223, DQ420224, DQ420225, DQ420226, DQ420227, DQ420228, DQ420229, DQ420230, DQ420231, DQ420232). The genes were then subcloned into the pMD19-T (simple) plasmid and further subjected to sequencing and bioinformatics analyses.

### 2.4. Analysis of feIFN-ω Gene and Protein Characteristics

Homology and phylogenetic tree analysis of the feline IFN-ω genes isolated in this study were analyzed using DNASTAR and MEGA7 software. In addition, the characteristics of the feIFN-ω genes and proteins were analyzed by several online bioinformatics software programs: signal peptide cleavage sites were analyzed by the SignalP 3.0 server at http://www.cbs.dtu.dk/services/SignalP-3.0/; phosphorylation sites were analyzed by the NetPhos3.1 server at http://www.cbs.dtu.dk/services/NetPhos/; N-glycosylation sites were analyzed by the NetNGlyc1.0 server at http://www.cbs.dtu.dk/services/NetNGlyc/; O-glycosylation sites were analyzed by the YinOYang1.2 server at http://www.cbs.dtu.dk/services/YinOYang/; subcellular localization was analyzed by the TargetP 1.1 server at http://www.cbs.dtu.dk/services/TargetP; transmembrane regions were analyzed by the TMHMM 2.0 server at http://www.cbs.dtu.dk/services/TMHMM/; antigen epitopes and hydrophobicity were analyzed by the BepiPred 1.0 server at http://www.cbs.dtu.dk/services/BepiPred-1.0/; and secondary and three-dimensional structures were predicted by SOPMA at https://npsa-prabi.ibcp.fr/cgi-bin/npsa.

### 2.5. Soluble Expression of Recombinant feIFN-ω

In this study, the genes encoding feIFN-ω that were isolated by RT-PCR were subcloned as a KpnI and BamHI-generated (New England Biolabs, MA, USA) gene fragment into the prokaryotic soluble expression plasmid pCold-TF, giving rise to recombinant pCold-feIFN-ω. After that, the recombinant plasmid was transformed into *E. coli* BL21 (DE3) competent cells and validated by PCR and sequencing analyses, thus generating the recombinant *E. coli* strain pCold-feIFN-ω/BL21. We then used sodium dodecyl sulfate-polyacrylamide gel electrophoresis (SDS-PAGE) analysis to determine the optimal conditions for expression of the recombinant feIFN-ω in pCold-feIFN-ω/BL21 cells via induction by isopropyl β-D-thiogalactoside (IPTG, Sigma, St. Louis, MO, USA). Briefly, for optimization of the IPTG concentration, the recombinant *E. coli* strain was grown in Luria–Bertani broth (Sigma, St. Louis, MO, USA) supplemented with 100 μg/mL ampicillin at 37 °C until the optical density at 600 nm was approximately 0.5. Then, IPTG was added at final concentrations of 0.4 mmol/L, 0.6 mmol/L, 0.8 mmol/L, 1.0 mmol/L, or 1.2 mmol/L, and the cultures were continually cultivated for another 6 h. After centrifugation at 12,000× *g* for 10 min, the cell pellets were lysed and analyzed by 12% SDS-PAGE. To determine the optimal induction time, the recombinant strain was induced by the optimized final concentration of IPTG for 4 h, 6 h, 8 h, 10 h, and 12 h. Following centrifugation and cells lysis, the proteins were again analyzed by 12% SDS-PAGE.

### 2.6. Determination of the Antiviral Activity of feIFN-ω

The fusion protein expressed by the pCold-feIFN-ω/BL21 bacteria cells was subjected to cleavage by the 3C protease (Sigma, St. Louis, MO, USA). The target recombinant feIFN-ω protein was then purified using Ni^2+^ affinity chromatography columns according to the manufacturer’s instructions, followed by confirmation analysis using SDS-PAGE. The purified feIFN-ω protein was stored at −80 °C until use. VSV was used as a virus model to evaluate the antiviral activity of the recombinant feIFN-ω via the in vitro microdose cytopathic effect inhibition assay (MCIA) according to the method described previously [12,26] with slight modifications. Briefly, 100 μL of the purified feIFN-ω sample was serially diluted ten-fold in DMEM containing 10% FBS and transferred to confluent F81 cell monolayers in 96-well cell culture plates, and then incubated at 37 °C in 5% CO_2_ for 18 h. F81 cells without IFN treatment were used as a control group. After incubation, VSV (MOI = 1) was prepared as described above, added into the 96-well plate, and incubated for 8-12 h until the CPE of the cells in the viral control group reached 100%. Next, the culture medium was removed, and the cells were stained with 0.2% crystal violet in 20% ethanol at 37 °C for 30 min. The cells were then destained with 0.1% acetic acid in 50% ethanol at 37 °C for 5 min before determining the absorbance of each well at 595 nm. Each sample was performed with eight biological replicates and three technical replicates. The antiviral activity of the IFN was calculated as the method previously described, which is expressed as units per milligram according to the ratio of IFN titer and protein concentration [27]. In parallel, the INTERCAT IFN antiviral drug (Toray Industries, Tokyo, Japan) was used as an IFN treatment control. In addition, we determined the species-specific antiviral activity of the recombinant feIFN-ω by conducting MCIAs using VSV in F81, Vero, MDCK, MDBK, and PK-15 cells. Broad-spectrum antiviral activity of the recombinant feIFN-ω was determined by conducting MCIAs using VSV, FCoV, CPV, BVDV, and PEDV.

### 2.7. Statistical Analysis

The results are shown as mean ± SEM (*n* = 3) for three independent experiments. Statistical analyses were performed using GraphPad Prism V5.0 software. Tukey’s multiple comparison tests and one-way analysis of variance (ANOVA) tests were used to analyze the significance of the differences between groups: * *p* < 0.05; ** *p* < 0.01; and *** *p* < 0.001.

## 3. Results

### 3.1. Identification of the Novel Feline IFN-ω Genes

**Figure 1:**
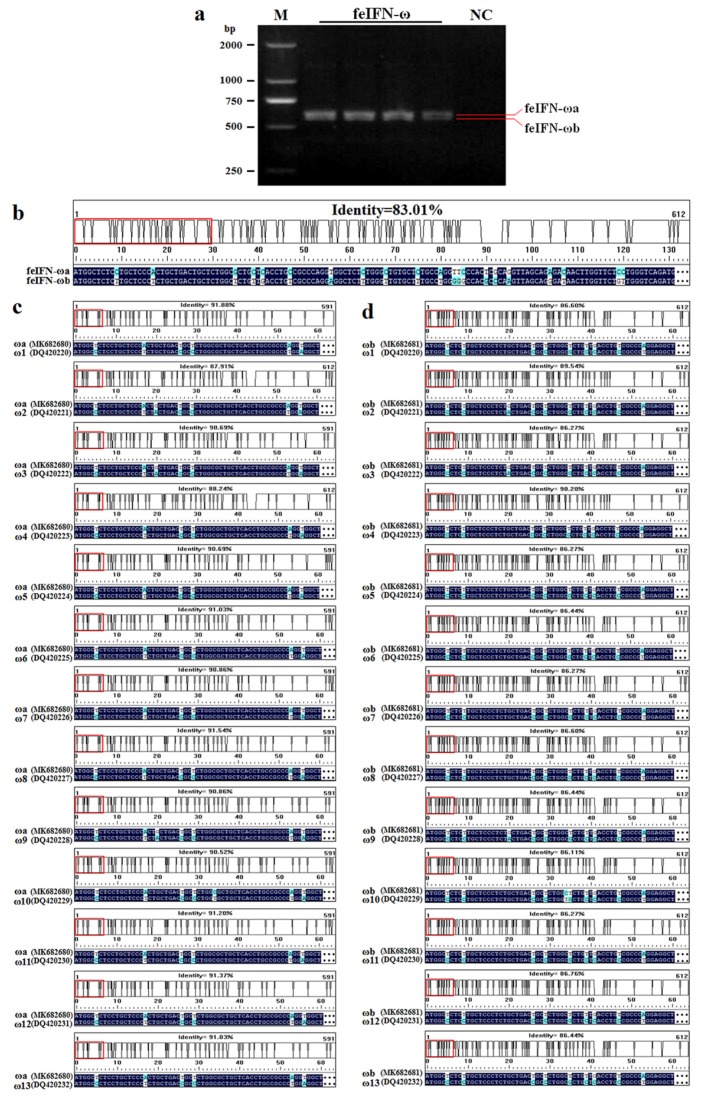
Identification of novel feline interferon omega (feIFN-ω) genes in splenic lymphocytes of cats. (**a**). Two genes, encoding feIFN-ωa and feIFN-ωb, were amplified by RT-PCR. M: DNA marker; NC: negative control. (**b**). Nucleotide sequence homology between the feIFN-ωa and feIFN-ωb genes. (**c**). Nucleotide sequence homology between the feIFN-ωa gene and the 13 known subtypes of feIFN-ω genes published in the GenBank. (**d**). Nucleotide sequence homology between the feIFN-ωb gene and the 13 known subtypes of feIFN-ω genes. Red box means comparison length unit.

We first collected splenic lymphocytes from cats infected with VSV and treated with poly(I:C) simultaneously. We then performed RT-PCR assays using a pair of degenerate primers to identify two genes encoding feIFN-ω, referred to as feIFN-ωa and feIFN-ωb (Figure 1a). These two novel genes were deposited in the GenBank with accession numbers MK682680 and MK682681. Following nucleotide sequence homology analysis, we found that the feIFN-ωa gene (MK682680), with a size of 591 bp, shared a maximum nucleotide sequence homology of 91.88% (91.6% amino acid homology) with the 13 known subtypes of feIFN-ω (feIFN-ω1 to feIFN-ω13) genes published in the GenBank (Figure 1c). The feIFN-ωb gene (MK682681), with a size of 612 bp, shared a maximum sequence homology of 90.20% (89.4% amino acid homology) with the 13 known subtypes of feIFN-ω (Figure 1d). The sequence homology shared between the feIFN-ωa and feIFN-ωb genes was only 83.01% (Figure 1b), indicating that the two genes identified in this study were novel feIFN-ω genes.

### 3.2. Phylogenetic Tree Analysis of the Newly Identified Feline IFN-ω Genes

**Figure 2:**
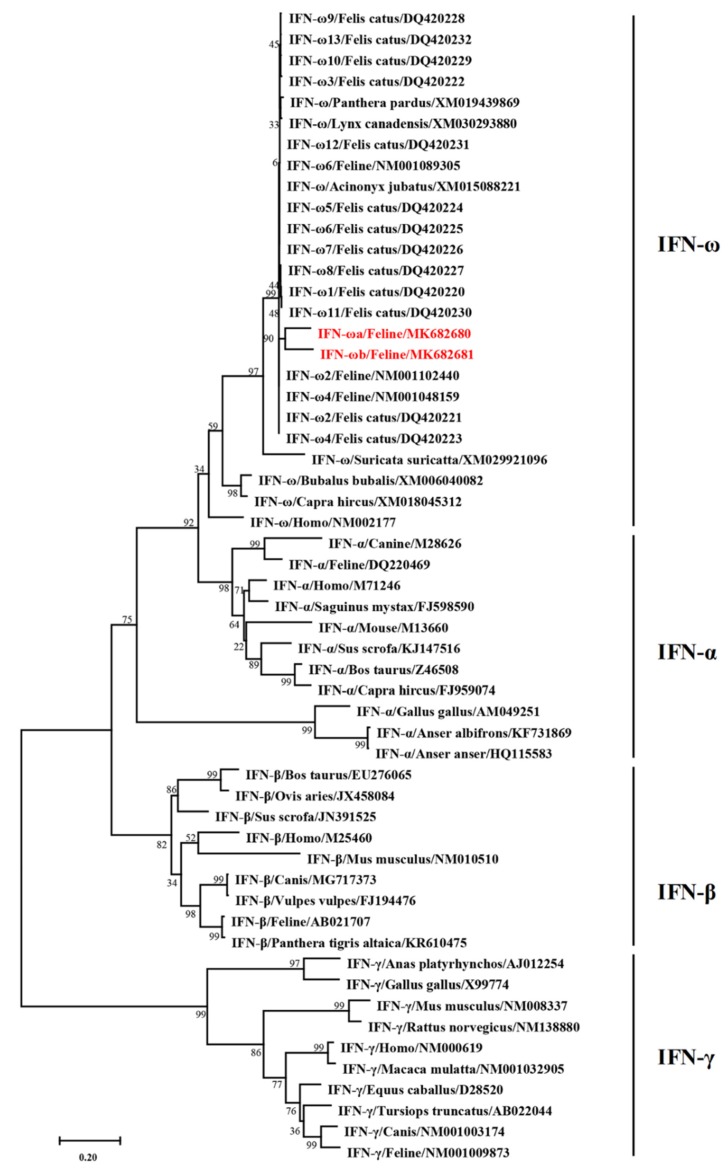
Phylogenetic analysis of nucleotide sequences of the feIFN-ωa and feIFN-ωb was performed with the maximum likelihood method using MEGA 7 software, compared with other IFNs found in cats and other animal species. The IFNs marked in red were cloned in this study.

We constructed a phylogenetic tree comparing the feIFN-ωa/ωb genes identified in this study with other IFN gene sequences published in GenBank from different animals using the software DNASTAR (Megalign) and MEGA7. The results showed that the feIFN-ωa/ωb genes belonged in the type I IFN family, but that their evolutionary relationship to the known feline IFN-ω genes already published in the GenBank (Figure 2) was distant, indicating that the feIFN-ωa and feIFN-ωb genes, identified for the first time here, are new subtypes of feline IFN-ω.

### 3.3. Bioinformatics Analysis for the Novel Feline IFN-ω Proteins

**Figure 3:**
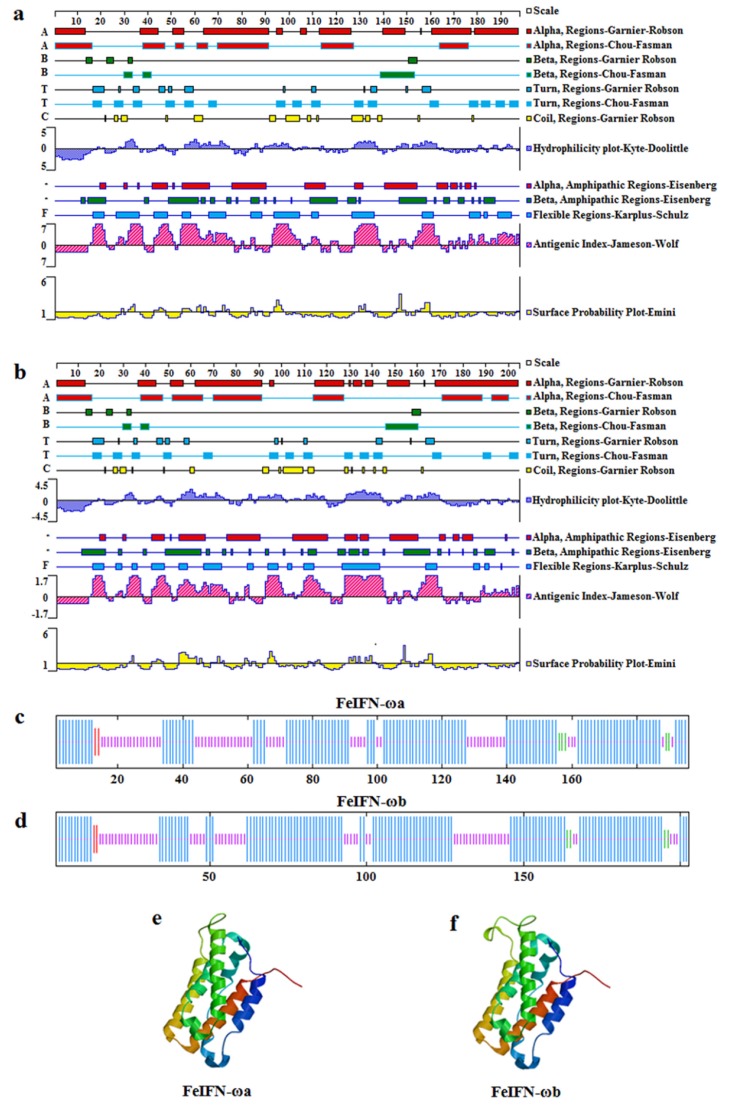
Prediction of antigen epitopes, hydrophobicity, secondary structure, and three-dimensional structure of the newly identified feline IFN-ω proteins. (**a**) The predicted antigen epitopes and hydrophobicity of feIFN-ωa. (**b**) The predicted antigen epitopes and hydrophobicity of feIFN-ωb. (**c**) The predicted secondary structure of feIFN-ωa. (**d**) The predicted secondary structure of feIFN-ωb. (**e**) The predicted three-dimensional structure of feIFN-ωa. (**f**) The predicted three-dimensional structure of feIFN-ωb.

The characteristics of the novel feline IFN-ω proteins (feIFN-ωa and feIFN-ωb) were analyzed using several online bioinformatics software programs, including the identification of potential signal peptide cleavage sites, N-glycosylation sites, O-glycosylation sites, phosphorylation sites, subcellular localization, and transmembrane regions. The results from these detailed analyses are displayed in Table 1. In addition, we also used online software algorithms to predict antigen epitopes, hydrophobicity, and the secondary and three-dimensional structures of the feIFN-ωa and feIFN-ωb proteins. As shown in Figure 3, the antigen epitopes of feIFN-ωa (predicted by BepiPred 1.0 software) were located at amino acids residues 19–23, 27–34, 66, 70–74, 96–107, 128–138, 158–162, 179–182, and 192-186 (Figure 3a), and the antigen epitopes of feIFN-ωb were located at amino acid residues 19–23, 27–33, 70–76, 96–107, 128–145, 165–169, and 200–203 (Figure 3b). The maximum hydrophobicity of feIFN-ωa was 1.92, and the minimum hydrophobicity was −2.27. The maximum hydrophobicity of feIFN-ωb was 2.27, and the minimum hydrophobicity was −2.36. The secondary structure of the two feIFN-ω proteins was predicted using SOPMA software and revealed that feIFN-ωa contained 62.24% alpha helix, 2.55% beta sheet, and 34.18% irregular curl structures (Figure 3c), whereas feIFN-ωb contained 65.52% alpha helix, 1.97% beta sheet, and 31.53% irregular curl structures (Figure 3d). The three-dimensional structures of feIFN-ωa and feIFN-ωb were predicted with SWISS-MODEL software and are shown in Figure 3e,f, respectively.

### 3.4. Soluble Expression and Purification of the Newly Identified feIFN-ω Proteins

**Figure 4:**
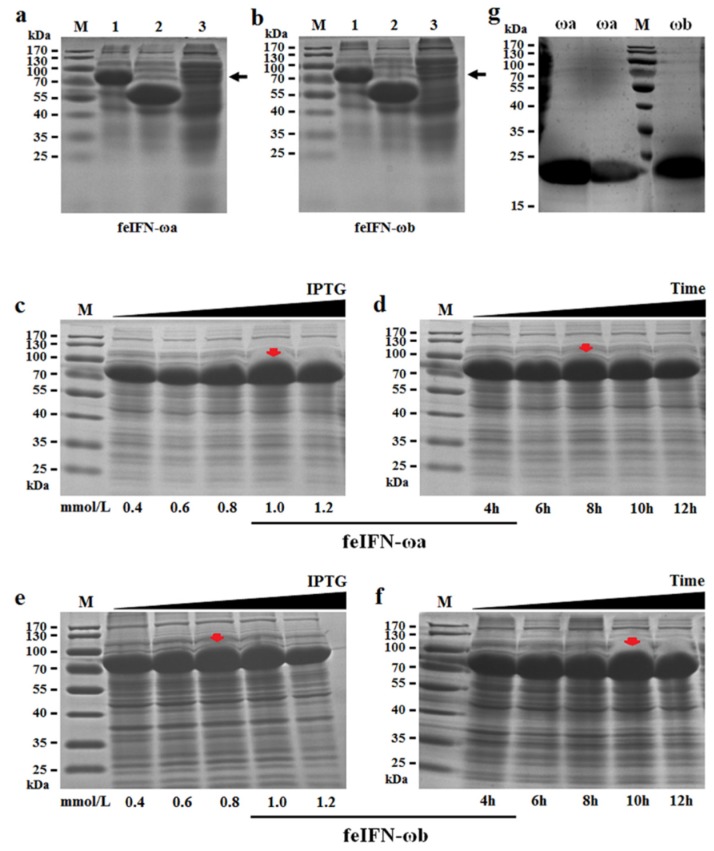
Expression and purification of recombinant feIFN-ω proteins from *E. coli*. (**a**,**b**) The fusion proteins were expressed from pCold-feIFN-ωa/BL21 (**a**) and pCold-feIFN-ωb/BL21 (**b**) in their soluble forms. M: Protein Marker; Lane 1: fusion protein rfeIFN-ωa+TF (**a**) and fusion protein rfeIFN-ωa+TF (**b**); Lane 2: TF tag expressed by pCold-TF/BL21; Lane 3: noninduced pCold-feIFN-ωa/BL21 (**a**) and noninduced pCold-feIFN-ωb/BL21 (**b**). (**c**,**d**) Optimization of the IPTG concentration (**c**) and induction time (**d**) conditions for expression of the rfeIFN-ωa protein from the pCold-feIFN-ωa/BL21 *E. coli*. (**e**,**f**) Optimization of the IPTG concentration (**e**) and induction time (**f**) conditions for expression of the rfeIFN-ωb protein from the pCold-feIFN-ωb/BL21 *E. coli*. (**g**) The rfeIFN-ωa and rfeIFN-ωb proteins were purified by His-tag Ni^2+^ affinity column chromatography. The black arrow represents the target fusion protein and red arrowhead represents the target fusion protein expressed under the optimized condition.

The genes encoding feIFN-ωa and feIFN-ωb were subcloned into the prokaryotic soluble expression vector pCold-TF and then transformed into *E. coli* BL21 competent cells, thus generating the recombinant protein expressing *E. coli* strains pCold-feIFN-ωa/BL21 and pCold-feIFN-ωb/BL21. After inducing their expression with IPTG, the rfeIFN-ωa (Figure 4a) and rfeIFN-ωb (Figure 4b) proteins fused with the trigger factor (TF) of approximately 57 kDa and were primarily expressed in their soluble forms. Subsequently, we optimized the expression conditions of the rfeIFN-ω proteins from the pCold-feIFN-ωa/BL21 and pCold-feIFN-ωb/BL21 *E. coli* strains. Our observations indicated that the optimal induction conditions for rfeIFN-ωa protein expression were an IPTG concentration of 1.0 mmol/L (Figure 4c) and an induction time of 8 h (Figure 4d), and the optimal induction conditions for rfeIFN-ωb protein expression were an IPTG concentration of 0.8 mmol/L (Figure 4e) and an induction time of 10 h (Figure 4f). After that, the fused proteins (rfeIFN-ωa/ωb+TF) were purified by His-tag Ni^2+^ affinity column chromatography and subjected to cleavage using the 3C protease. The cleaved proteins were then purified by His-tag Ni^2+^ affinity column chromatography once again, after which we collected the purified recombinant rfeIFN-ωa and rfeIFN-ωb proteins with molecular weights of approximately 21 kDa and 22 kDa, respectively (Figure 4g).

### 3.5. Determination of the Antiviral Activities of Recombinant feIFN-ωa and feIFN-ωb

**Figure 5:**
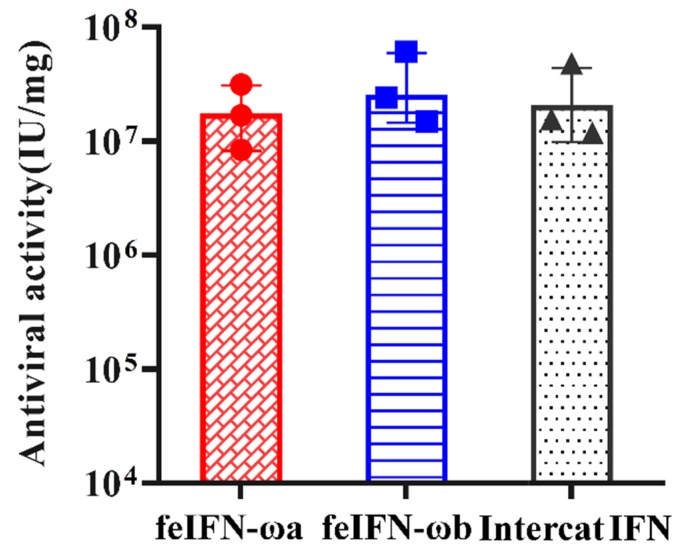
The antiviral activities of the recombinant feIFN-ωa and feIFN-ωb proteins against vesicular stomatitis virus (VSV) propagated on F81 cells, as determined by microdose cytopathic effect inhibition assays. INTERCAT IFN is an antiviral drug that we used as a positive control, and F81 cells untreated with the IFNs were used as negative control. The dots, square and triangle in the bars mean each test value of the three repetitions, respectively.

We determined the antiviral activity of rfeIFN-ωa and rfeIFN-ωb using microdose cytopathic effect inhibition assays (MCIAs) and VSV as the viral model that was propagated on F81 cells. As shown in Figure 5, both rfeIFN-ωa and rfeIFN-ωb demonstrated good antiviral activity similar to the INTERCAT IFN (feline IFN antiviral drug) positive control. However, no antiviral effect in negative control groups (cells untreated with the IFNs) was detected.

### 3.6. Species-Specific Antiviral Activities of Recombinant feIFN-ωa and feIFN-ωb

**Figure 6:**
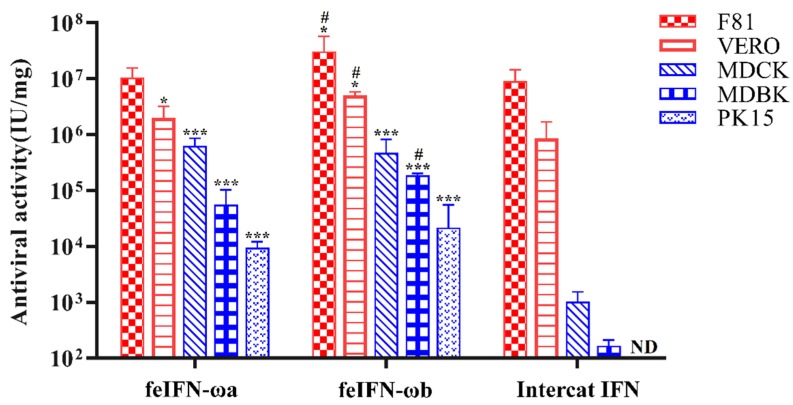
The species-specific antiviral activities of rfeIFN-ωa and rfeIFN-ωb using INTERCAT IFN for comparison. Bars represent the mean ± SEM for each group. Significant differences (* *p* < 0.05; *** *p* < 0.001) were observed when comparing the rfeIFN-ωa/rfeIFN-ωb groups to the INTERCAT IFN group. Comparison of the rfeIFN-ωb group and rfeIFN-ωa group also indicated significant differences (# *p* < 0.05).

We then tested whether the antiviral activities of rfeIFN-ωa and rfeIFN-ωb were species-specific. To address this question, we performed MCIAs with VSV propagated in several different cell lines from different animal species, including F81 cells (cat), Vero cells (monkey), MDCK cells (dog), MDBK cells (cattle), and PK-15 cells (pig). As shown in Figure 6, the purified recombinant feIFN-ωa and feIFN-ωb proteins showed antiviral activity in both homologous animal cells (F81 cells) and heterologous animal cells (Vero, MDCK, MDBK, and PK-15 cells) in vitro. The antiviral activity of the rfeIFN-ω proteins in heterologous animal cells was significantly stronger than that of the INTERCAT IFN control. Although the INTERCAT IFN displayed high antiviral activity in F81 and Vero cells, it had significantly weak antiviral activity in MDCK and MDBK cells, and no antiviral activity in PK-15 cells. Furthermore, the antiviral activity of rfeIFN-ωb was better than that of rfeIFN-ωa in homologous and heterologous animal cells, but especially in homologous animal cells that originated from cats. However, no antiviral activity was detected in cells’ control groups (cells untreated with the IFNs).

### 3.7. Broad-Spectrum Antiviral Activities of feIFN-ωa and feIFN-ωb

**Figure 7:**
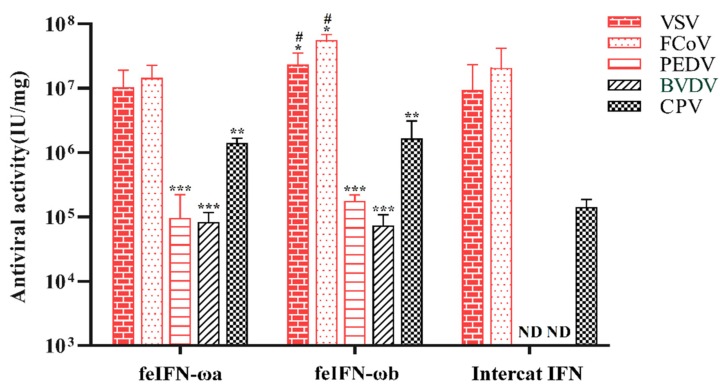
The broad-spectrum antiviral activities of rfeIFN-ωa and rfeIFN-ωb using INTERCAT IFN for comparison. Bars represent the mean ± SEM for each group. Significant differences (* *p* < 0.05; ** *p* < 0.01; *** *p* < 0.001) were observed when comparing the rfeIFN-ωa/rfeIFN-ωb groups to the INTERCAT IFN group. A comparison of the rfeIFN-ωb group and rfeIFN-ωa group also indicated significant differences (# *p* < 0.05).

Next, we wanted to investigate whether the antiviral activities of the recombinant feIFN-ωa and feIFN-ωb proteins were also broad-spectrum. We performed MCIAs using a wide range of different viruses, including VSV, FCoV, CPV, BVDV, and PEDV. As shown in Figure 7, rfeIFN-ωa and rfeIFN-ωb both displayed in vitro antiviral activity against VSV, FCoV, CPV, BVDV, and PEDV. Their antiviral activities were especially strong against FCoV and VSV. In contrast, the antiviral activity of rfeIFN-ωb against FCoV and VSV was significantly stronger than that of rfeIFN-ωa. No notable differences were observed between rfeIFN-ωa and rfeIFN-ωb with CPV, BVDV, or PEDV. In addition, the antiviral activity of rfeIFN-ωb against FCoV and VSV was significantly stronger than that of INTERCAT IFN, and the antiviral activities of both rfeIFN-ωa and rfeIFN-ωb against CPV, BVDV, and PEDV were significantly higher than that of INTERCAT IFN. In fact, we observed no antiviral activity against BVDV or PEDV by INTERCAT IFN. Unsurprisingly, there was no antiviral activity detected in cells’ control groups (cells untreated with the IFNs).

## 4. Discussion

Currently, cats are continuing to become more and more common as household pets. We know of a wide variety of viral infections that can seriously endanger the health of pet cats, such as FeLV, FIV, FIPV, FCoV, and feline calicivirus (FCV). Interferon represents a promising potential therapeutic agent that can effectively treat pet cats infected with these viruses. In this study, we identified two new genes encoding feline IFN-ω (feIFN-ωa and feIFN-ωb) in the spleen lymphocytes of cats. Following sequence homology analysis, we found that feIFN-ωa and feIFN-ωb shared maximum nucleotide sequence homologies of 91.88% and 90.20%, and maximum amino acid homologies of 91.6% and 89.4% with the 13 previously published subtypes of feline IFN-ω, respectively. In addition, phylogenetic tree analysis of IFNs in cats and other species constructed using neighbor-joining analysis revealed that feIFN-ωa and feIFN-ωb do belong to the type I IFN family, but their evolutionary relationship to known feline IFN-ω genes was distant, indicating that feIFN-ωa and feIFN-ωb were indeed new subtypes of feline IFN-ω. We deposited these two genes into the GenBank with the accession numbers MK682680 (feIFN-ωa) and MK682681 (feIFN-ωb), thus enriching the IFN-ω data submitted to the GenBank [12].

In this study, the characteristics of the feIFN-ωa and feIFN-ωb proteins, such as signal peptide sequences, signal peptide cleavage sites, phosphorylation sites, glycosylation sites, antigen epitopes, hydrophobicity, and transmembrane regions were analyzed using bioinformatics to provide better theoretical guidance for the functional study of these proteins. The mature proteins, with normal biological activity, were formed only after the signal peptide sequence was removed from the precursor protein, thus allowing them to be secreted outside the cell membrane [28,29]. We used online software to predict that the signal peptide sequence of the feIFN-ωa/ωb proteins consists of 23 amino acid residues, and that the signal peptide cleavage site is located between residues Gly23 and Cys24. The results indicated that the recombinant feIFN-ωa and feIFN-ωb proteins could be expressed in vitro in their soluble forms. Glycosylation is an important post-translational modification process that can affect the antigenic determinants, charge properties, enzymatic properties, and thermal stability of proteins. Similar to previous reports for the 13 known feIFN-ω subtypes [12], we observed no N-glycosylation sites present in feIFN-ωa or feIFN-ωb. However, our analyses predicted nine potential O-glycosylation sites in feIFN-ωa and six potential O-glycosylation sites in feIFN-ωb, which is different from previous reports on the other known IFN-ω subtypes [6]. Studies have shown that glycosylation sites can play an important role in determining the activity of IFNs [30,31]. For example, glycosylated IFN-ω has been observed to be markedly more potent than non-glycosylated IFN-ω against hepatitis C virus, BVDV, yellow fever virus, and West Nile virus, with even more superior effects than IFN-α, IFN-β, and IFN-γ [6,30].

Insect/baculovirus expression systems are one of the most effective eukaryotic expression systems for preparing feline IFNs. These expression systems are capable of making post-translational modifications, such as glycosylation, which allows proteins to fold correctly, thus producing highly active and stable IFNs [32,33,34,35,36]. However, the application of these systems is limited by drawbacks, such as low yield of IFN protein and complicated operation requirements [37]. Therefore, *E. coli* expression systems are still the most widely used prokaryotic expression system for protein production with their characteristics of simple procedures, large-scale production, and low cost [38,39,40]. Generally, recombinant IFN is produced by *E. coli* expression systems in an insoluble inclusion body form that has not been modified or folded correctly, resulting in the loss of its biological activity [41,42]. To obtain a soluble and biologically active IFN protein, the inclusion bodies need to be denatured and then renatured, which is a time-consuming, laborious process [40]. In this study, we selected the prokaryotic soluble expression system pCold-TF to prepare recombinant feIFN-ωa and feIFN-ωb. The pCold-TF system is a highly efficient soluble expression system with a His-tagged TF that allows the expressed protein to be effectively modified, folded, and secreted into the cytoplasm [43,44]. Following the construction of recombinant *E. coli* strains and induction by IPTG, the rfeIFN-ωa/ωb proteins were expressed in their soluble forms and analyzed via SDS-PAGE. We optimized expression conditions for the rfeIFN-ωa and rfeIFN-ωb proteins and purified them using His-tag Ni^2+^ affinity column chromatography. Both proteins showed antiviral activity against VSV in microdose cytopathic effect inhibition assays using F81 cells. Our results provide a basis for further studies into the development of rfeIFN-ωa and rfeIFN-ωb as therapeutic agents for viral infections. Meanwhile, our data provide evidence that the pCold-TF expression system can be used to produce IFN with full bioactivity.

Recombinant feline interferon-ω (rfeIFN-ω) was the first licensed immunomodulator for use in treating viral infections in cats, especially FIV and FeLV infections [17,18,45]. Furthermore, rfeIFN-ω also exhibits therapeutic effects against other feline viral infections, such as FCV, feline parvovirus, and feline herpesvirus-1 [45], as well as viruses that originate in other animals, such as foot-and-mouth disease virus, influenza virus, BVDV, VSV, PRV, and rotavirus [6,12,18]. In this study, our results demonstrated that both rfeIFN-ωa and rfeIFN-ωb had antiviral activity in homologous animal cells (F81 cells, cat) and heterologous animal cells (Vero cells, monkey; MDBK cells, cattle; MDCK cells, dog; PK-15 cells, pig), indicating that rfeIFN-ωa and rfeIFN-ωb have broad cross-species antiviral activity in vitro, similar to previously published findings in MDBK and MDCK cells [12]. In contrast, the antiviral activities of rfeIFN-ωb in homologous and heterologous animal cells were better than that of rfeIFN-ωa. Intriguingly, the rfeIFN-ωa and rfeIFN-ωb proteins exhibited antiviral activity in MDCK cells, despite the absence of IFN-ω in canines [46,47]. We speculate that different IFNs with different physiological functions are likely responsible for the difference of results obtained in our study compared to other published studies. Furthermore, analysis of the broad-spectrum antiviral activities of rfeIFN-ωa and rfeIFN-ωb revealed that they were effective against VSV, FCoV, PEDV, BVDV, and CPV. Out of those five viruses, antiviral activity was strongest against VSV and FCoV. No significant differences in antiviral activity against CPV, BVDV, or PEDV were observed between rfeIFN-ωa and rfeIFN-ωb. However, the overall broad-spectrum antiviral activity of rfeIFN-ωb was significantly stronger than that of rfeIFN-ωa.

Recently, combination antiviral therapy has become a common practice in treating feline viral infections due to pharmacokinetics and the short half-life of IFN alone [48,49]. There are many published studies focused on the combination of IFN-ω with other therapeutic agents, such as chemotherapeutic agents [50], IFN-α [51], and ribavirin [6], suggesting this is an attractive strategy to use against viral infections. We further evaluated the antiviral effects of rfeIFN-ωa and rfeIFN-ωb combined with feline IL-18 against VSV and FCoV in F81 cells. Our results revealed that the in vitro antiviral activity of combination therapy was significantly increased compared to that of rfeIFN-ωa or rfeIFN-ωb alone, indicating that rfeIFN-ω combination therapy may represent a more potent option than monotherapy for treating viral infections in cats; however, this hypothesis requires further exploration in vivo. In summary, the rfeIFN-ωa and rfeIFN-ωb obtained in this study exhibit significant broad-spectrum antiviral activity in both homologous and heterologous cells in vitro, particularly rfeIFN-ωb, suggesting a promising candidate for the development of an effective therapeutic agent against viral infections in cats and other animals.

## 5. Conclusions

In this study, two new subtypes of feline IFN-ω (ωa and ωb) were identified and characterized. They shared a maximum nucleotide sequence homology of 91.88% and 90.20% and a maximum amino acid homology of 91.6% and 89.4% with the 13 previously known subtypes of feIFN-ω, respectively. We analyzed the characteristics of feIFN-ωa and feIFN-ωb in detail using bioinformatics followed by soluble expression and optimization of induction conditions in *E. coli*. Our data showed that purified recombinant feIFN-ωa and feIFN-ωb had broad-spectrum antiviral activity in homologous and heterologous animal cells, suggesting they are candidates for the development of effective therapeutic agents to be used against viral infections in pet cats. And, our research is underway to systematically evaluate the effectiveness of the two novel feIFNs as therapeutics agent for cat viral infections in vivo. In addition, the reported feIFN-ω sequences will enrich the IFN data submitted to GenBank.

## Figures and Tables

**Table 1:** Bioinformatics analysis for the two novel feline interferon omega (feIFN-ω) proteins.

IFN	Signal Peptide Cleavage Sites ^a^	No. of Phosphorylation Sites ^b^	No. of Glycosylation Sites ^c^	Subcellular Localization ^d^	Transmembrane Region ^e^
IFN-ωa	Gly23-Cys24	15	*N*-glycosylation sites (0) *O*-glycosylation sites (9)	82.1% extracellular 7.2% intracellular 9.6% mitochondrion	Intracellular
IFN-ωb	Gly23-Cys24	13	*N*-glycosylation sites (0) *O*-glycosylation sites (6)	84.3% extracellular 9.2% intracellular 7% mitochondrion	Intracellular

^a^ signal peptide cleavage sites were analyzed by SignalP 3.0 Server at http://www.cbs.dtu.dk/services/SignalP-3.0/; ^b^ phosphorylation sites were analyzed by NetPhos3.1 Server at http://www.cbs.dtu.dk/services/NetPhos/; ^c^ *N*-glycosylation sites were analyzed by NetNGlyc1.0 at http://www.cbs.dtu.dk/services/NetNGlyc/ and *O*-glycosylation sites were analyzed by YinO Yang1.2 at http://www.cbs.dtu.dk/services/YinOYang/; ^d^ subcellular localization was analyzed by TargetP 1.1 Server at http://www.cbs.dtu.dk/services/TargetP; ^e^ transmembrane region was analyzed by TMHMM Server v. 2.0 at http://www.cbs.dtu.dk/services/TMHMM/.
